# Adjustable Single-Osteotomy Fibular Free Flap for Anterior Mandibular Defects in Irradiated Head and Neck Cancers—A Case Series

**DOI:** 10.3390/jcm14061953

**Published:** 2025-03-13

**Authors:** Chien-Chung Chen, Ting-Han Chiu, Abdurezak Ali Mohammed, Hsiang-Shun Shih

**Affiliations:** 1 Department of Plastic and Reconstructive Surgery, E-DA Hospital, Kaohsiung City 824, Taiwan; ed113814@edah.org.tw (T.-H.C.); shih0825@ms37.hinet.net (H.-S.S.); 2 College of Medicine, I-Shou University, Kaohsiung City 824, Taiwan; 3 ALERT Hospital, Ababa 1000, Ethiopia; abdialim@yahoo.com

**Keywords:** osteotomy, fibula, mandibular reconstruction, radiotherapy, head and neck cancer

## Abstract

**Objective**: Reconstructing the anterior mandible in patients with irradiated and contracted soft tissues remains challenging despite advances in computer-assisted design and three-dimensional printing. Unpredictable soft-tissue changes reduce the effectiveness of these technologies. This paper explores an alternative using a single-adjustable-osteotomy fibula flap technique. **Methods**: A retrospective study was performed on patients with anterior segmental mandibular defects due to recurrent tumors, secondary reconstruction, or osteoradionecrosis and previously received radiotherapy who represented the highest risk of soft tissue complexity while limiting the utility of computer technology. All patients underwent mandible reconstruction using the adjustable, single-osteotomy fibula method, which eliminated the need for computer-assisted design. We evaluated the effectiveness and outcome. **Results**: From 2016 to 2023, 11 patients were included in this study. The median patient age was 58 (ranging 49–65) years. Included patients had either recurrent tumors (n = 6), secondary reconstruction needs (n = 3), or mandibular osteoradionecrosis (n = 2). No complete flap failures occurred. Five of six patients with recurrent cancer required two skin island fibular flaps for intraoral and external defect repair. One patient experienced partial skin paddle loss requiring an additional free flap, and another had plate exposure requiring removal after bone union was achieved. **Conclusions**: The adjustable single-osteotomy fibula flap technique offers a reliable alternative for anterior mandibular reconstruction in complex cases. This approach demonstrates advantages in surgical simplicity and flexibility while maintaining acceptable outcomes. However, careful patient selection and consideration of defect extent remain crucial for success.

## 1. Introduction

Segmental mandibulectomy inflicts serious functional impairment and destruction to appearance, which remains a considerable technical challenge even to the most experienced reconstructive surgeons [1,2]. The anterior mandible is crucial for supporting the dentition and the tongue. If the reconstruction is postponed, the remaining bilateral mandibular stump ends often collapse inwards, resulting in a narrowed and retrusive jaw, known as the “Andy Gump deformity” [3]. This would render future mandibular reconstructions unattainable as the intricate three-dimensional structure and the vital functional significance are difficult to restore.

Whilst various new reconstructive techniques have emerged over the years, the free fibular flap still reigns as the gold standard in mandibular reconstructions for its superior bone stability, durability to the irradiation effect, and aesthetic outcomes [4,5,6]. Recent advances in computer-assisted surgical planning (CASP) and custom-made cutting guides have revolutionized this field, offering enhanced precisions, reduced operative times, and decreased complication rates [7,8,9,10]. These technological innovations have proved particularly valuable in anterior mandibular reconstruction, where its unique complex architecture demands upmost accuracy.

In actual clinical practice, however, obstacles may occur when applying CASP to complex cases involving tumor recurrence, extensive scarring, or irradiated tissues. The unpredictable nature of tissue transformation after surgical resection and irradiation often hinders accurate preoperative planning [11,12]. Critical intraoperative variables—including the extent of scar tissue resection, tumor margins, and soft tissue contracture—can all substantially alter the final characteristics of the resultant mandibular defect predicted through the preoperative images [11,12,13]. Moreover, the depletion of recipient vessels and the compromised soft tissue envelopes frequently necessitate intraoperative improvisations as the initial surgical plan and cutting guides may fail to adapt to those circumstances [12]. The preparation of serial models according to possible alterations is both laborious and costly.

The authors were thus inspired to develop a flexible, single-osteotomy fibula flap technique to function as either a backup strategy or an alternative when digital planning is infeasible. We selected a specific patient population presenting factors of high potential technical inaccessibility in standard CASP designs and replaced them with our proposed V-shaped designs. This study aimed to assess the clinical outcomes and possible complications associated with this streamlined technique in anterior mandibular reconstructions.

## 2. Materials and Methods

### 2.1. Patient Inclusion and Exclusion Criteria

A retrospective review of patients with head and neck squamous cell carcinoma (HNSCC) who underwent sequential oncologic resection, flap reconstruction, and adjuvant radiotherapy from 2016 to 2023 at E-Da Hospital in Kaohsiung, Taiwan was conducted, with approval from the institutional review board obtained under the number EMRP-112-135. This case series study consisted of patients with anterior segmental mandibular defects strictly confined to the area between the two angles who required free fibular reconstructions due to one of the following three conditions: recurrent tumor necessitating additional resection, secondary or delayed mandibular reconstruction, or mandibular osteoradionecrosis (ORN). During initial consultation, all patients were given the choices of conventional computer-simulation-assisted strategy or the proposed adjustable-osteotomy model, with thorough explanation of the benefits and possible limitations regarding both approaches. The authors specifically selected patients who wished to avoid the complex simulation process and were not willing to risk intraoperative reconstruction guide misfit to receive our alternative method while those with high expectations for masticatory function restoration or prioritizing superior aesthetic outcomes were otherwise excluded. All of the selected patients underwent the adjustable-osteotomy, free fibular-based mandibular reconstruction and received regular clinic follow-up postoperatively. We then assessed the surgical outcomes, complications, and efficiency.

### 2.2. Surgical Planning and Technique

**Figure 1:**
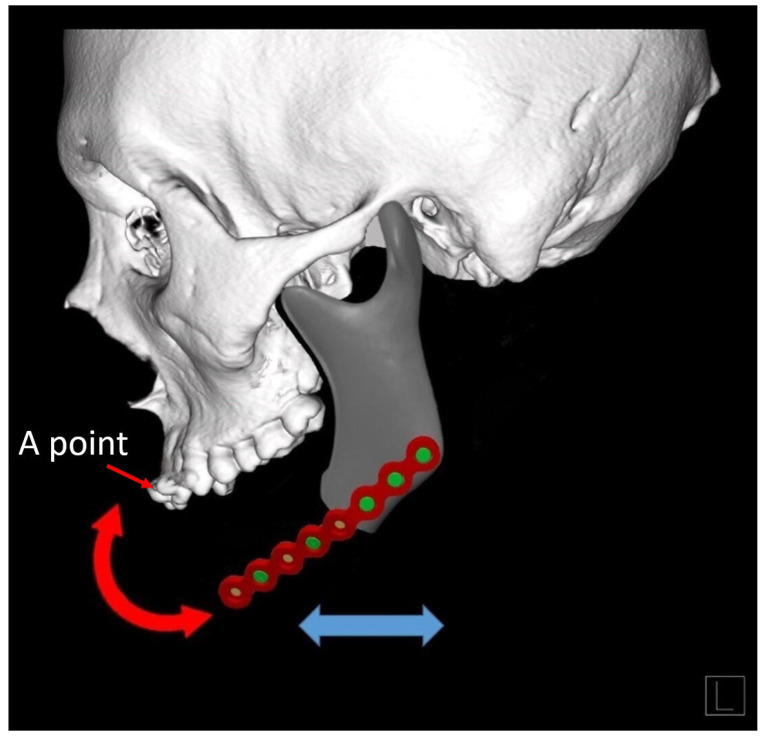
A point was defined as a reference for maintaining the projection and midline of the neo-mandible. The bending plate could be used as a guide and freely adjusted for the best chin position.

**Figure 2:**
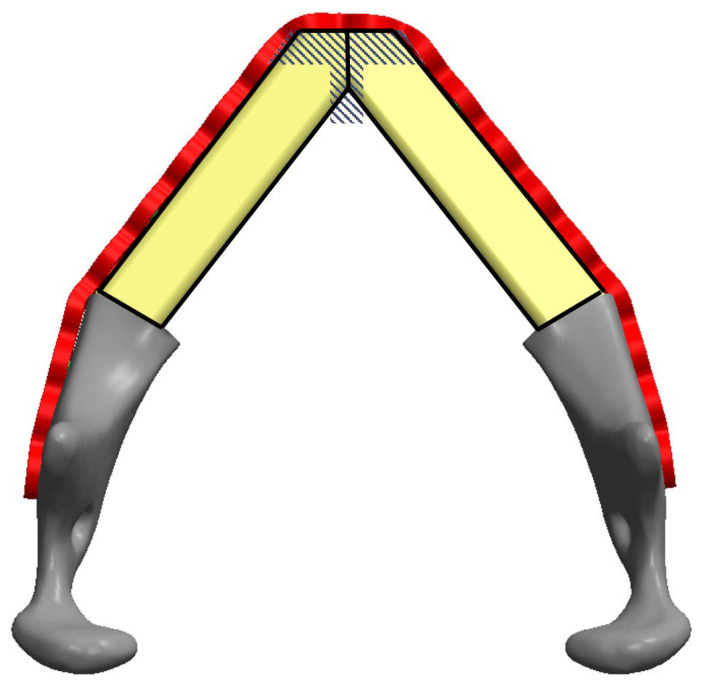
Demonstration of fitting the fibular bone segments to the manually bent reconstruction plate. The center contacting bone ends were blunted to avoid the chin being at an acute angle (area of dashed lines).

After resecting the contracted tissues around the mandible, the margins of the remaining mandibular stumps were released for optimal position. Measurements for the fibular bone design were then performed directly on site according to the actual defect. A central reference, Point A, located either at the midpoint of the upper central incisors or the most anterior-central maxillary ridge in edentulous patients, was established as a midline orientation guide for the placement of the most prominent anterior-central part of the neo-mandible design for proper chin projection (Figure 1). The distances from this central point to the bilateral mandibular defect ends were measured, then referenced to modify a 2.0/2.3 mm mandible reconstruction plate (Stryker, Leibinger, Portage, MI, USA) for simulation of the malleable metal template of our neo-mandible design shape. This V-shaped manually bent plate was then fitted to the inferior border of the bilateral mandibular defects. Manual modification of the plate was then performed to balance the chin projection and soft tissue coverage. The position of the plate was adjusted to achieve the optimal maxillomandibular relationship and facial profile while the center of the plate was angled bluntly to avoid a sharp appearance and to reduce pressure at the overlying chin skin (Figure 2). After confirming the ideal position, the plate was temporarily secured with screws.

**Figure 3:**
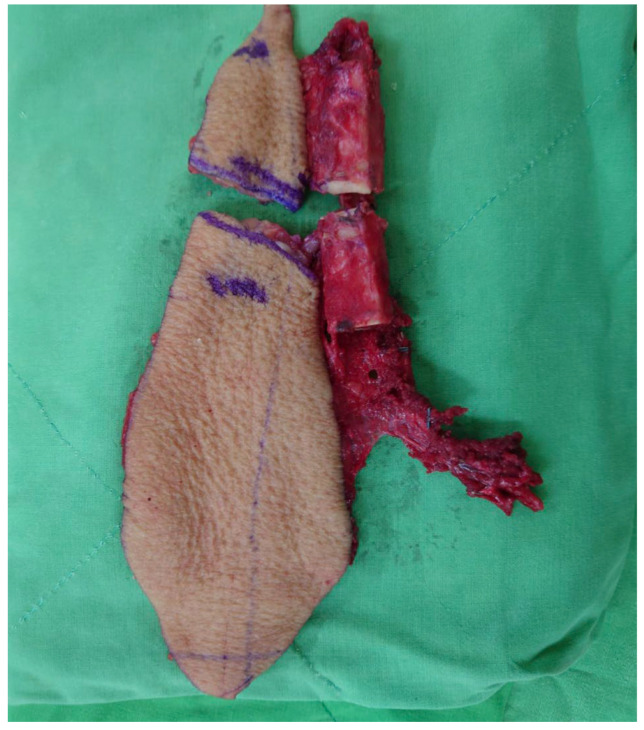
A fibular flap was harvested with two skin islands and two bone segments.

**Figure 4:**
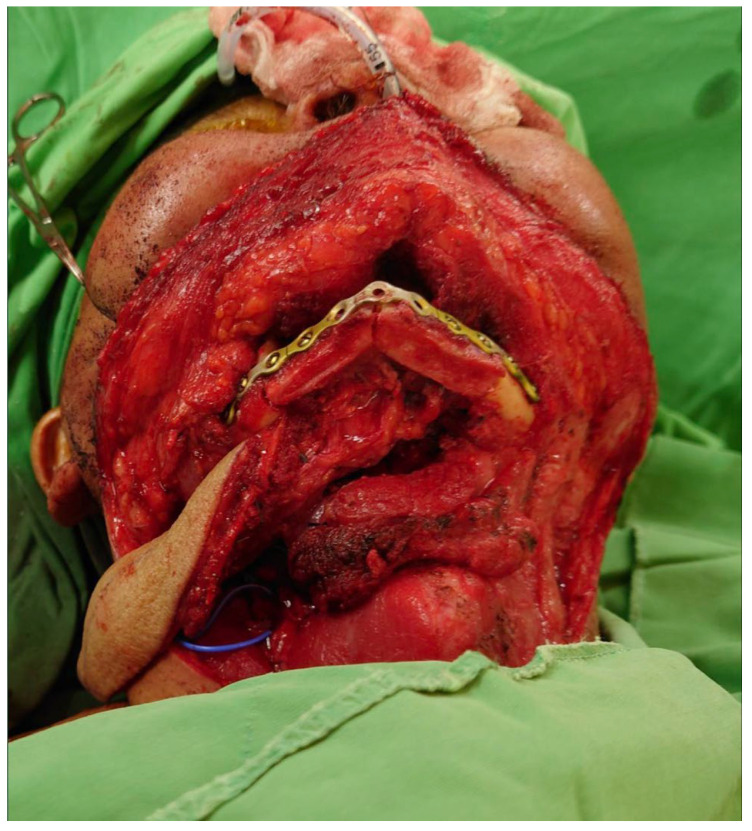
The fibular flap was shaped then fixed alone with the plate to achieve the best contact and smooth border. The skin flap was well sutured to replace the oral mucosal and chin skin defects.

The required lengths of the two fibular bone segments were measured along the inner surface of the reconstruction plate from the defect margins to the anterior-central aspect of the plate. A free fibular osteocutaneous flap was harvested via standard technique. The fibular bone was osteotomized according to the previous measurements (Figure 3). The contacting ends of these two bone segments were then contoured to attain an optimal fit with the manually bent plate at a blunt angle for achieving a more natural aesthetic outcome (Figure 4). Final adjustments of segment angle, length, chin contour, and soft-tissue requirements were made before definitive fixation. An implantable Doppler device was placed for flap monitoring. The donor site was closed either primarily or with a skin graft.

## 3. Results

From 2016 to 2023, 38 mandibular reconstructions were performed using free fibula flaps. Patient demographics were listed in Table 1. Eleven patients with previous radiotherapy who met the designated criteria of potential difficult reconstruction (six patients with recurring tumors that invaded their mandibles, two patients with ORN, and three patients undergoing secondary mandibular reconstructions for unrepaired mandibular defects from previous surgeries) were included in this study. The median age was 58 years (49–64).

The average lengths of the two fibular bone segments were 4.5 cm and 4.2 cm. No flap failure was observed in our series. Five of the six patients with recurrent cancer needed a two-skin-island fibular osteocutaneous flap for intraoral mucosal and external skin defect repair. One patient of recurrent oral cancer who underwent a single-skin-flap fibular reconstruction of the mandibular defect suffered skin paddle partial loss, and received another free anterior lateral thigh flap for the resurfacing and coverage of the exposed bone. One patient experienced oral mucosal wound disruption with plate exposure 3 months postoperatively, yet the wound healed seamlessly following the removal of the plate as the bone graft had already achieved proper union. The follow-up time ranged from 1.5 to 7 years postoperatively, with a mean of 2.82 years.

### 3.1. Case Presentation

#### 3.1.1. Case I

**Figure 5:**
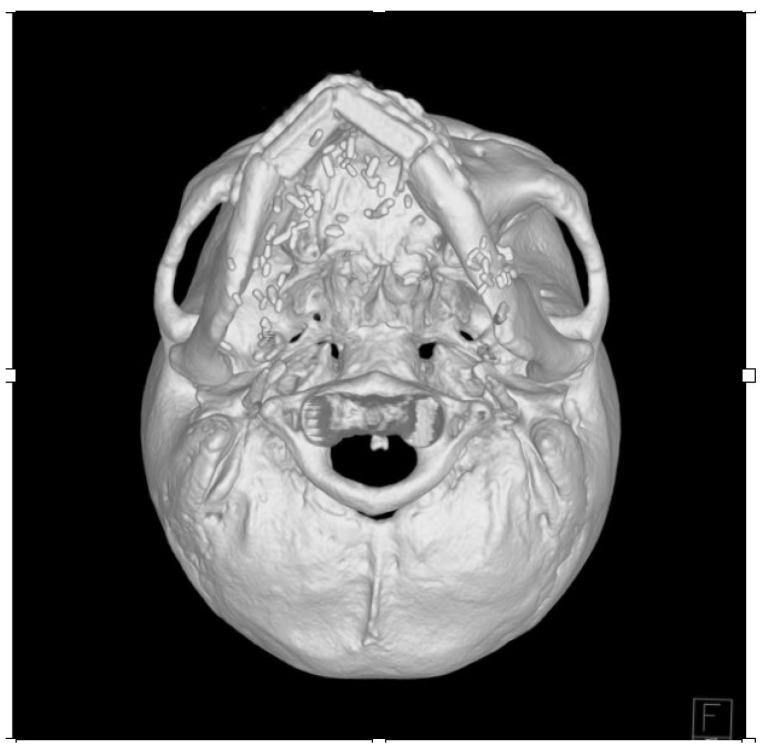
Postoperative computed tomography scan of achieving smooth mandibular contour.

**Figure 6:**
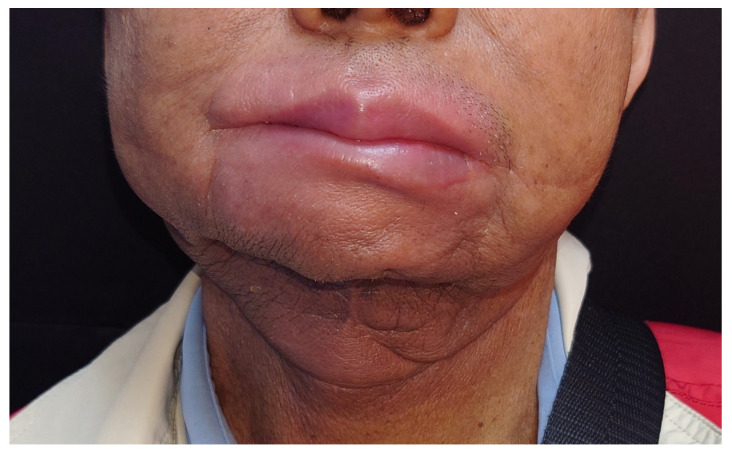
Patient was able to retain a stable chin support and jaw contouring postoperatively.

This 64-year-old patient of irradiation history experienced oral cancer recurrence. After composite resection, there was a need to address the mandibular bone defect and through-and-through defect over his mouth floor mucosa and chin skin. The reconstruction plate was bent and adjusted to achieve optimal chin position and served as a guide for the subsequent fibular design. A single V-shaped osteotomy design was adopted for the fibular bone, along with two-skin islands, which were then well shaped and fixed accordingly (Figure 4). We successfully achieved the long term result of a smooth chin contour and stable bony support (Figure 5 and Figure 6).

#### 3.1.2. Case II

**Figure 7:**
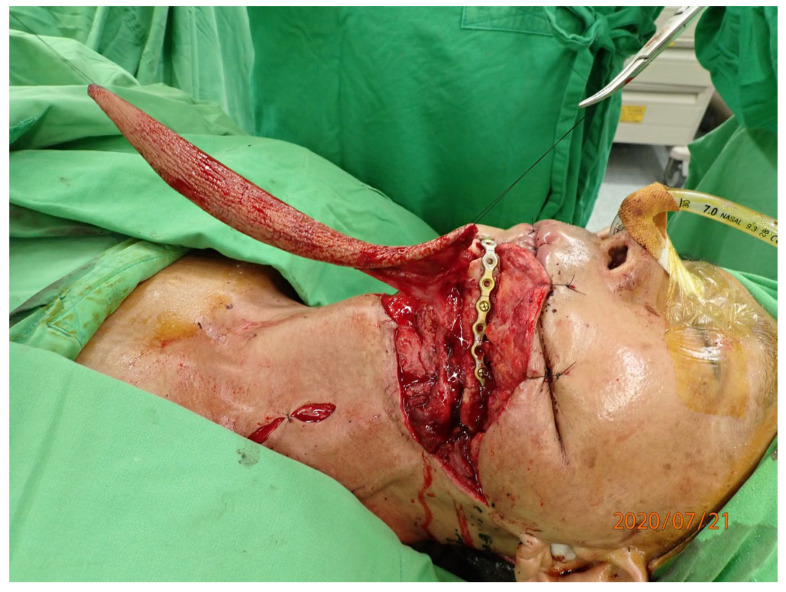
A well-designed fibula flap can be used to replace the defect in the mandibular bone and neck soft tissue.

**Figure 8:**
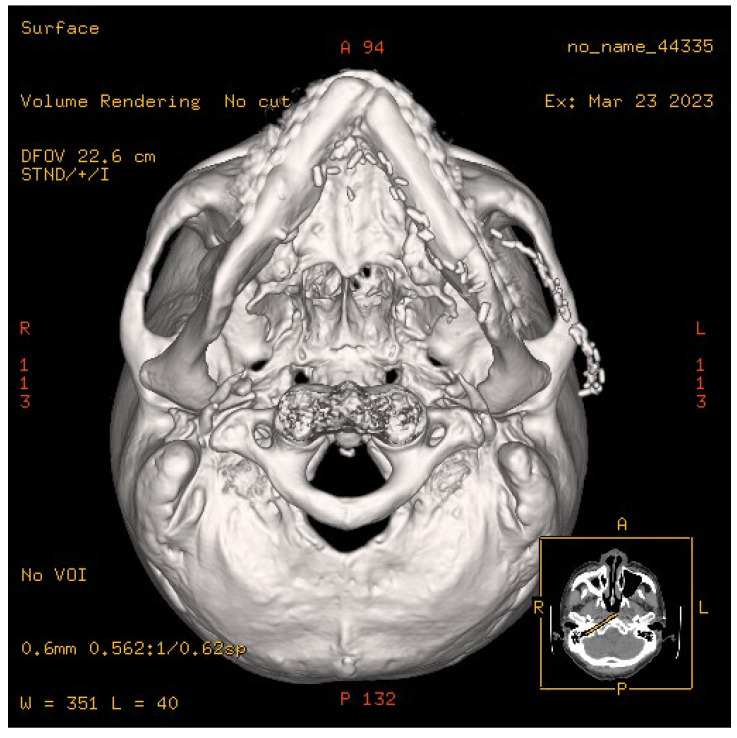
The anterior mandibular defects of different lengths could be well adjusted and repaired through a single-osteotomy-designed fibula.

**Figure 9:**
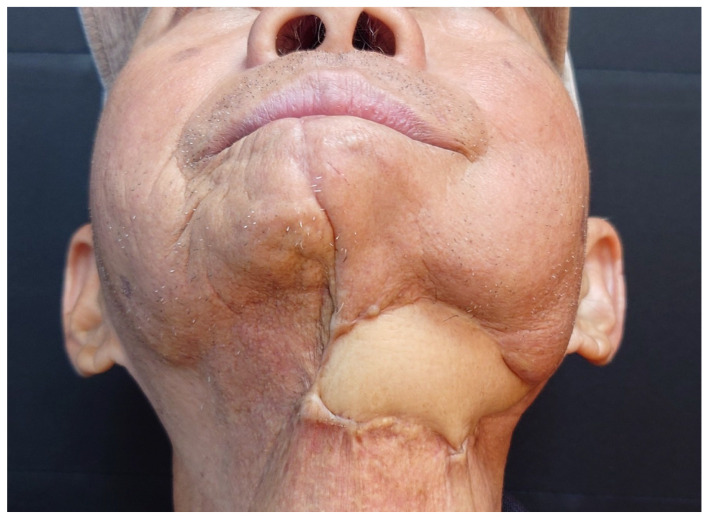
The patient’s neck wound was well healed, and there was improvement in neck movement.

This 56-year-old patient suffered from mandibular ORN. After serial sequestrectomies, the patient developed severe neck contracture with movement limitation and a chronic ulceration over the neck. The meticulous excision and release of scarring tissues was performed and no additional mouth floor mucosal defect was created during the process. The V-shaped fibular bone and its skin paddle smoothly repaired the mandibular bone and neck skin defects (Figure 7). The uneven length of the mandibular defect could be fairly adjusted and a stable bone framework and proper jaw contour could be achieved (Figure 8). The troublesome neck ulcer healed smoothly, and the patient reported improved neck movement postoperatively (Figure 9).

## 4. Discussion

Vascularized autogenous bone grafting remains the gold standard for the reconstruction of anterior mandibular defects to avoid sequelae of uncontrolled drooling, tongue drop, airway obstruction, and conspicuous disfiguration following deformity [4,5,6]. Compared to alloplastic reconstructions, free fibular flaps result in less hardware exposure and failure rates, especially in those receiving adjuvant radiotherapy and those with mandibular defects at the central parts [15]. However, it is a particular technical challenge to perform such reconstruction secondarily since the collapsed lateral mandibular stumps would lead to chin retrusion and the loss of bony support. Also, compared to primary reconstruction, scar contracture or irradiated soft tissue further heightens surgical complexity [16].

The advent of computer-assisted surgical planning, custom-made cutting guides, and three-dimensional plate printing technology has led to optimized reconstruction precision, the predictability of surgical results in vascularized fibular flap reconstructions, and a shift in the current trend towards being more prosthodontically driven [7,8,9,17]. Nevertheless, in terms of recurrent cancer ablations, irradiated patients, and secondary reconstructions, these technologies have become less adaptive to the unpredictable tissue flexibility due to the disposition of residual bone stumps after muscle retraction, reduced soft tissue elasticity after scar contracture, and scarce recipient vessel choices to fit the pedicle orientation [13]. Without a reliable actual guide for the intended original mandibular segment design, surgeons would seek a mirror image from the contralateral side, reimaging from previous computed tomography, or even artificial intelligence simulation from human databases [13]. These factors further amplify possible errors in virtual planning and increase the chance of intraoperative manual adjustment or sometimes the inevitable discarding of the simulated models, bringing great technical distraught to surgeons and deep disappointment to patients that expects better results given the high costs. Some authors have attempted to establish protocols to overcome these challenges, including comprehensive vascular imaging evaluation [18,19] and multiple backup simulation plans [11]. Regardless of the revision protocols, surgeons still resort to the concept of fitting fibular bone segments along a template plate as the only known and most reliable guide under such complicated circumstances.

Our study design uniquely tested a simple reconstruction method without the need for CASP in patients of high simulation-failure risk factors induced from our previous clinical experiences. We enrolled three specific patient groups including those with recurrent tumors, those with mandibular ORN, and those needing secondary reconstruction and successfully validated that our innovative approach could still manage in the demanding conditions surgeons face when digital technology fails or is inaccessible. For recurrent tumors, the main challenge lies in the uncertainty of preoperative defect estimation as the final ablation extent and dimension is affected by the distorted anatomical structures and mixed tissue layers, as well as being determined by the pathological margins under microscopic exams of frozen sections. In ORN cases, the major issue is to determine how extensive the sequestrectomy should be as no current modern imaging technology can precisely identify all areas of bone viability prior to an actual resection [20]. The shape and osteotomy of the fibular flap required may thus greatly differ from the preoperative planning conducted solely from image simulation, which could cause serious stress and panic for surgeons facing unexpected cutting guide fails or inset difficulty, especially under the pressure of ischemia time. However, our intraoperative measurement approach combined with manually bent plate guiding offers a simple, easy-to-grasp design concept and yet allows greater flexibility in coping with such situations calmly.

It is of utmost importance to note that all of our patient demographics experience significant scarring and tissue contraction resulting from prior surgical procedures and radiotherapy. The loss of tissue elasticity makes it difficult to predict the actual dimension of the bone gap even after scar resection. Also, these patients commonly display scarring that extends beyond the surgical site, impacting the intraoral mucosa and previous flap even to the cheek and neck skin. The aggressive excision of scar tissue could enhance the anatomic repositioning of the mandible but may carry more risks than benefits, especially in cases of ORN and secondary reconstruction. First, the extensive excision of scars may result in the formation of new complex soft-tissue defects in the already fibrotic mucosa, skin, or previous flaps. Plus, the mandibular ramus or condyle regions may also be surrounded by scarring, rendering a complete release unattainable. Lastly, in patients who have undergone reconstructive surgery and radiation therapy, the depletion of recipient vessels requires thoughtful assessment since it could greatly influence the availability, selection, and inset planning of the flap [12,18]. As a consequence, we advocate for a judicious and conservative approach to managing scar tissue in these patient populations as this may mitigate technical complexity, particularly if patients are amenable to suboptimal outcomes.

Our proposed single, adjustable osteotomy along with the V-shaped fibular bone design also offers technical flexibility in anterior mandibular reconstructions. Conventionally, the use of U-shaped three-segment fibular bone design remains the mainstream approach along with computer-assisted planning cutting guides serving as valuable assistance [21,22,23,24]. Nevertheless, when the dimensions of the bone defect are altered, it becomes inevitable to simultaneously adjust both the lengths and osteotomy angles of all three fibula segments in order to attain optimal maxillomandibular relationships. If they wish to adhere to the original cutting guide and to retain the bilateral end-segment lengths while maintaining the integrity of the central segment and osteotomy angles, a patient could end up with a less-desired squared or shortened chin.

The human mandible has been shown to have a highly constant symphysis width of about 27 mm and angles of the arch of around 120° on each side, unaffected by age or sex [25]. Therefore, the center segment would be inevitably under 3 cm in order to fit in the U-shape three-segment arch design, risking malperfusion or bone absorption [25,26]. Moreover, it requires considerable clinical experience to ensure aesthetic precision if one wishes to maintain the U-shape contour via the traditional hand saw cutting and plate fixation method in such short and small bone segments. Multiple attempts and osteotomies in such small bone segments may prolong the procedure time and damage the blood supply [26,27]. Unlike the double-osteotomy U-shape design, a single-osteotomy V-shaped design is relatively straightforward in clinical practice and does not require a steep learning curve for surgical team members, thereby potentially reducing the operative time and iatrogenic complications. Another advantage of this design lies in its enhanced flap pedicle flexibility. The simplified geometry facilitates a more effective adaptation to access recipient vessels afar, even when the redirection of the flap is mandated to reach vessels on the contralateral side. This method can also be easily applied to cases of uneven bilateral mandibular defects (as demonstrated in case II). It effectively maintains the midline and projection of the neo-mandible without the need for special facilities.

In the Chinese Han population, the inter-canine distance from the mandible is merely an approximate 25–28 mm [28]. Therefore, for Asians in whom chins are usually smaller and less prominent, a two-segment V-shape design could avoid perfusion issues yet still retain acceptable appearance as the overlying soft tissue also provides some volume bulk.

For patients of HNSCC stemming from betel nut chewing who were mostly edentulous and relied on a soft-to-liquid diet prior to operation, the necessity of future dental prosthesis or implants was less significant than in patients of benign causes like ameloblastomas or the medication-related osteonecrosis of the jaw. Thus, restoring an acceptable contour in the appearance and retaining bony support to prevent drooling or chin deviation is the priority in such reconstructions and could be achieved fairly well with the simpler two-segment V-shape design compared to the intricate U-shape design.

However, this design poses the potential risk of increased pressure over the chin apex, leading to skin necrosis and plate exposure. Our modification utilizes a smoother plate contouring and trimming of the bone edges to form a blunt angle configuration at the apex, effectively reducing pressure on the overlying tissues. Throughout our series, no patients developed chin plate exposure or skin necrosis. Though one ORN patient did require plate removal due to intraoral mucosal wound disruption at three months post operation, satisfactory bone union had already been achieved prior to plate removal.

Furthermore, additional soft tissue coverage is frequently necessitated in reconstructing anterior mandibular defects after tumor ablations. The two-skin-island fibula flap is a valuable technique for addressing both intraoral and skin defects in such circumstances [21]. Preoperative computed-tomography angiography may assist in identifying the locations of the perforators essential for designing the skin island flap and osteotomy [22,29], which significantly contributes to the successful implementation of the complex bone segments and dual skin paddles simultaneously. In our study, we performed a single osteotomy with two-skin paddles of the fibular flap to repair the coexisting mucosal and cutaneous tissue defects in recurrent tumor cases. The single-osteotomy design allows more working space for cutting the bone safely while effectively preserving the delicate perfusion from perforators to both the bone segment and the skin paddles. We observed no circulation compromise in those receiving the two-skin fibular flaps.

Our proposed single- and adjustable osteotomy fibular flap design serves as a reliable tool and salvage backup plan in certain difficult anterior mandibular reconstructions when CASP falls short or is unavailable. However, our study still had several limitations that warrant discussion. The relatively small sample size and highly selective patient cohort, especially including only those could accept suboptimal aesthetic results and could not afford virtual simulation planning, limit the generalizability of this approach. The absence of a control group further prevented us from making direct comparisons with other reconstruction techniques. The functional and aesthetic outcomes were also fairly subjective, being drawn from descriptions from the surgeon and patients. Further objective evaluation tools and standardized questionnaires should be implemented in future studies.

In our study, we attempted to minimize the use of double free flap reconstruction, yet we agreed that double free flaps remained important if one wishes to achieve adequate soft tissue coverage and optimal functional outcomes in more challenging cases presenting with severe chin contracture, significant soft tissue deficiency, or advanced tumor resection requiring extensive ablation. Based on our clinical experience, we recommend applying our technique to defects without extending beyond the midpoint of the mandibular body since wider defects may result in undesirable triangular appearances. For such extensive bone defects, we recommend incorporating additional segments, possibly utilizing a U-shaped design, to achieve more aesthetically pleasing results.

This study introduced a new perspective and innovation on anterior mandibular reconstruction; nonetheless, it requires additional research with larger patient cohorts, mandible rehabilitation data, and more thorough comparative analyses.

## 5. Conclusions

The effective management of soft tissues in mandibular defects with scar contracture is essential. A versatile surgical plan is vital for addressing the complexity and variability of intraoperative changes. An adjustable, single-osteotomy fibular flap reconstruction provides a flexible, simple, yet reliable alternative for specific patients with anterior mandibular defects.

## Figures and Tables

**Table 1:** Patient demographics.

No.	Age	Gender	Mandibular Defect		Length of Fibular Bone Segment	Fibular Skin Island		Complication	Follow-Up Time (Years)
			Types	Location ^#^		Paddle	Size (cm)		
1	65	Male	Secondary mandible reconstruction	CH	6 cm + 3 cm	Single	20 × 9		3
2	49	Male	Recurrent oral cancer	CL	3 cm + 3 cm	Single	20 × 8		2
3	65	Male	Recurrent oral cancer	LC	4 cm + 2 cm	Single	15 × 8		2
4	52	Male	Recurrent oral cancer	CL	3 cm + 3 cm	Two	12 × 7 8 × 5		1.5
5	50	Male	Recurrent oral cancer	LC	3 cm + 5.5 cm	Two	15 × 7 7 × 5		5
6	64	Male	Secondary mandible reconstruction	LCL	5 cm + 6.5 cm	Two	8 × 4 12 × 5		7
7	58	Male	Mandible ORN	LCL	3 cm + 5.5 cm	Single	11 × 5		3
8	58	Male	Secondary mandible reconstruction	LCL	5.5 cm + 5 cm	Single	15 × 8		2
9	64	Male	Recurrent oral cancer	LCL	3 cm + 3 cm	Two	13 × 7 3 × 5		1.5
10	64	Male	Recurrent oral cancer	LC	11 cm + 6 cm	Single	18 × 7	Skin paddle partial loss	2
11	49	Male	Mandible ORN	LCL	3.6 cm + 3.7 cm	Single	18 × 8	Oral wound dehiscence with plate exposure	2

^#^: Jewer classification (H: lateral defects including the condyle but that did not cross midline; L lateral defects without the condyle and that did not cross midline; C: defects consisting of the entire central segment containing the four incisors and the two canines) [14].

## Data Availability

Raw data can be retrieved upon request.
